# Approaches to use the WHO respiratory syncytial virus surveillance platform to estimate disease burden

**DOI:** 10.1111/irv.12667

**Published:** 2019-10-08

**Authors:** Richard Pebody, Jocelyn Moyes, Siddhivinayak Hirve, Harry Campbell, Sandra Jackson, Ann Moen, Harish Nair, Eric A. F. Simões, Peter G. Smith, Niteen Wairagkar, Wenqing Zhang

**Affiliations:** ^1^ Flu Surveillance Public Health England London UK; ^2^ Center for Respiratory Diseases and Meningitis National Institute for Communicable Diseases Johannesburg South Africa; ^3^ Global Influenza Program World Health Organization Geneva Switzerland; ^4^ Usher Institute of Population Health Research and Informatics University of Edinburgh Edinburgh UK; ^5^ Center for Global Health Colorado School of Public Health Aurora CO USA; ^6^ MRC Tropical Epidemiology Group London School of Hygiene and Tropical Medicine London UK; ^7^ Bill and Melinda Gates Foundation Seattle WA USA

**Keywords:** burden, respiratory syncytial virus, surveillance

## Abstract

The World Health Organization (WHO) recently completed the first phase of a RSV surveillance pilot study in fourteen countries (two to three in each WHO region) building on the Global Influenza Surveillance and Response System (GISRS). This active surveillance strategy had several objectives including understanding RSV‐related health burden in a variety of settings. A range of approaches can be used to estimate disease burden; most approaches could not be applied by participating countries in the WHO surveillance pilot. This article provides the recommendations made by WHO for strengthening and expanding the scope of the RSV surveillance in the next phase to enable burden estimation.

## 1 BACKGROUND

Respiratory syncytial virus (RSV), an acute respiratory viral infection which can result in severe disease and death particularly for young infants, is being increasingly recognized as an important cause of morbidity and mortality globally. Shi et al have estimated that globally RSV‐associated lower respiratory tract infection (LRTI) accounted for between 94 600 and 149 400 deaths annually. In addition, 33 million RSV‐associated LRTI resulted in 3.2 million hospital admissions.^1^ The authors also describe a higher burden in low‐ and middle‐income countries (LMICs) suggesting that these populations may benefit most from a future intervention. These estimates, however, have some limitations, particularly due to lack of data from several high burden areas including sub‐Saharan Africa and limited data in narrow age bands for younger children, where the burden is usually highest and in whom future interventions are likely to be targeted.

Recently, significant progress has been made in the development of a range of RSV vaccine candidates, in particular for the protection of infants via maternal immunization. In addition, new longer acting monoclonal antibodies that can protect children for several months are in late‐stage clinical development. These developments have ignited interest in this area. Accurate estimates of RSV‐related health burden including costs are needed to undertake economic evaluations comparing the cost‐effectiveness of alternative interventions.

Ahead of the potential availability of new RSV vaccines for pregnant women and young children and new generation monoclonal antibodies and to improve awareness of its importance among policy‐makers, health staff and the public, the World Health Organization (WHO) established an RSV surveillance pilot study in 2017 in fourteen countries (two to three in each WHO region) building on the Global Influenza Surveillance and Response System (GISRS), hereafter referred to as the pilot. The pilot had several objectives including contributing to understanding of the health burden of RSV‐related infection in a range of settings. This article reviews the potential approaches that can be used to estimate disease burden and explores their applicability to the WHO RSV surveillance based on the influenza platform.

## 2 DESCRIPTION OF WHO PILOT

The WHO RSV surveillance pilot (1) took place in fourteen countries ranging from high to low income and from tropical to temperate settings. The main aim was to assess whether global RSV surveillance could fit into the GISRS platform. A secondary aim was to assess the feasibility of collecting data points needed for burden estimates in participating countries. The focus of the pilot was to collect data to estimate the proportion of respiratory hospitalizations associated with RSV. In some settings, this was extended to outpatient or primary care settings. Many countries were successful in implementing the clinical and laboratory aspects of the surveillance including collection of the required sample of clinical specimens.

## 3 BURDEN OF DISEASE DUE TO RSV

The burden of disease can be characterized in several different ways. Burden on the health service is often quantified in terms of numbers of outpatient/primary healthcare consultations; emergency department attendances and hospital admissions. Burden on the population may also be described by the numbers of infections including short‐term morbidity and any longer‐term chronic consequences of infection plus deaths associated with infection.

Burden of disease estimates may also include the cost of diseases (either direct costs to the health service or including indirect cost to patients and carers due to work loss). For example, a study in Bangladesh demonstrated a median direct cost of USD 10 million of RSV‐associated hospitalizations among children <5 years of age in 2010 and an indirect cost of USD 3 million.^2^ Better description of the health burden due to RSV infection will assist policy‐makers to make informed decisions on the allocation of scarce health resources when considering new vaccines or other interventions for RSV disease.

Obtaining an accurate estimate of the disease‐specific healthcare burden can be challenging, as often the investigation, laboratory testing and reporting of infectious diseases such as RSV are incomplete or absent. RSV‐associated infection is known to be very common as shown with ad hoc age‐stratified serological studies, for example a study in Kenya indicated that 100% of the population are likely to be infected by 3 years of age.^3^ Only a proportion of these infections, however, will be symptomatic and a smaller proportion of them will present to health services. In a prospective cohort study in Finland, the average annual symptomatic RSV infection rate was 275/1000 in children <3 years of age over two RSV seasons, of whom 58% developed acute otitis media, and only 3% of those symptomatic were hospitalized.^4^ Outside of enhanced studies such as these, most of those with infections that contact the healthcare service including hospitalizations will remain untested for RSV. Use of routine surveillance data alone without enhanced RSV testing may thus significantly underestimate the burden of disease due to RSV‐associated LRTI on the healthcare system and on the health of the general population.

## 4 POTENTIAL METHODS TO MEASURE BURDEN OF DISEASE USING A SURVEILLANCE PLATFORM

A range of approaches, many deployed for influenza, can be used to estimate local‐, country‐ and international‐level RSV‐related burden in terms of numbers of cases and disease incidence either using established disease surveillance systems and vital statistics or through special studies. Their potential application in the context of the WHO surveillance pilot (1) is explored:

- *Method 1: Regression modelling using routine data sources*. This ecological approach uses national or large‐scale routine laboratory surveillance and administrative health service data that have been collected consistently over several years. It has been utilized in some countries to measure the burden of a wide range of infections including influenza and RSV.^5^, ^6^, ^7^ Based on weekly data on primary care consultations, hospitalizations and deaths due to ICD‐coded respiratory disease, the regression model attributes a proportion of the respiratory infection such as pneumonias and bronchiolitis to RSV (and influenza) using weekly laboratory data adjusting for key confounders such as meteorological conditions. This statistical approach requires seasonality in the infection of interest and health data availability over several years. The method has also been applied to derive international burden estimates for the 2009 influenza pandemic where parameter values were used to derive estimates for those countries where data were lacking.^8^ Those WHO pilot countries with access to such routine health data could potentially apply such methodology.
- *Method 2: Multiplicative modelling.* This approach has been applied to estimate influenza‐related mortality for the 2009 pandemic by multiplying age‐specific symptomatic infection attack rate and symptomatic case fatality rate from a range of income settings. With this method, a respiratory mortality multiplier was defined to consider differences in outcome according to setting. Simulation models with these parameters were then applied across all countries to derive global estimates of influenza‐related mortality.^9^ A similar approach has been used in a recently published modelling study to estimate the global hospital admission rate and mortality rate due to RSV‐related acute lower respiratory tract infection (ALRI) using information on the proportion of hospital ALRI admissions that were due to RSV and the case fatality ratio (CFR) for RSV‐ALRI.^1^ However, gaps in RSV knowledge remain. This article noted that very limited RSV hospital and mortality data were available from low‐income settings, particularly in sub‐Saharan Africa and the Eastern Mediterranean region. The WHO pilot surveillance aims to gather relevant hospital surveillance data that will strengthen burden estimates, particularly from low‐income settings.
- *Method 3: Data linkage.* Linkage of routine hospital administrative data to laboratory records has been used in developed settings such as Australia to better describe RSV epidemiology.^10^ This approach can provide more detailed information on patients with laboratory‐confirmed RSV, such as prevalence and type of underlying risk factors, length of stay and likelihood of different outcomes. Estimates of RSV‐related hospital admissions can be derived, considering differences in laboratory testing practice by age and other key risk factors. These individual‐level data allow more accurate cost information to be generated.^10^, ^11^ To our knowledge, few such studies have been undertaken in LMIC settings and the approach is unlikely to be applicable in most countries participating in the WHO pilot surveillance due to lack of electronic administrative databases in hospitals or primary care settings.
- *Method 4: Enhanced surveillance/prospective cohorts*. The establishment of prospective population cohorts, with the comprehensive systematic collection of epidemiological information and biological samples from participants, allows RSV epidemiology and burden to be studied more directly—both in the community and in primary and secondary care settings. The Influenza and RSV in Infants Study (IRIS) is a multi‐country prospective study in four low‐income settings and was set up to assess the frequency of influenza and RSV in children <1 year of age who have been hospitalized and to ascertain predictors of more severe disease.^12^ Prospective population‐based studies have also been created, including household studies to describe the burden and transmission of RSV in both low^13^‐ and high‐income settings.^4^ Such studies can also provide information on key parameters needed for realistic transmission models and provide the opportunity to gather information on quality of life in young RSV‐positive children, information which at present is largely lacking. At present, there are only a very limited number of such studies as they are resource intensive to establish and maintain and thus tend to cover very limited periods.
- *Method 5: Simple, rapid assessment approach*. This methodology has been used to estimate the national burden of influenza using surveillance data from sentinel sites and by applying several adjustments to obtain a national estimate.^14^, ^15^, ^16^ This approach is simple and involves using the severe acute respiratory illness (SARI)/LRTI rate from an established sentinel hospital network, adjusting for key risk factors for pneumonia (such as malnutrition, indoor air pollution and HIV infection) at the provincial or district level and multiplying by the RSV proportion positive from hospital surveillance to derive the RSV‐associated SARI rate. This was intended to be the approach applied in the WHO pilot.

## 5 EXPERIENCES IN COLLECTING HOSPITAL AND COMMUNITY MORBIDITY DATA IN THE RSV SURVEILLANCE PILOT AND THE WAY FORWARD IN THE NEXT PHASE

The results of the pilot were reviewed at a face‐to‐face meeting (Bangkok, 2018) organized by WHO. Some countries were only able to implement a convenience sampling strategy and relied on attending physicians to collect samples and baseline clinical information. Most of the participating pilot countries lacked data from routine hospital admissions, catchment populations and administrative registers to estimate the burden of RSV‐associated hospitalization stratified in different age bands and disaggregated by week or month. This meant none of the burden estimation methods outlined above could be applied at this first stage of pilot—including method 5.

## 6 FUTURE PLANS

Building on the learning from the pilot, an extension phase is planned and coordinated by WHO. Going forward in the next phase of the WHO RSV surveillance, countries agreed at a further face‐to‐face meeting (Kathmandu, 2019) on the importance of prioritizing hospitalization burden associated with RSV in young children. The importance of defining a minimum data set was identified and that additional efforts would be required to collect harmonized burden‐related data based on surveillance. Most countries proposed the need to set up systems to collect healthcare attendance data more systematically as part of surveillance that could inform policy‐making in circumstances where no data from any of the recognized approaches outlined earlier are available in that country. In the absence of resources for special disease burden studies, a tiered approach towards disease burden estimation using surveillance data was proposed for the second phase of the WHO surveillance. Table 1 shows the data source for the burden‐related variables that are needed to measure the burden estimate, the adjustment factors, the preconditions to avoid bias and the limitations and caveats to each of the estimate. The burden estimates are grouped based on increasing levels of complexity of collecting data from a sentinel surveillance platform. Tier 1 groups burden estimates related to proportions of hospitalization associated with RSV. Tier 2 groups population‐based incidence estimates of hospitalization, ICU‐based burden and mortality estimates including case fatality ratio and proportion of hospital deaths due to RSV. The third tier refers to national extrapolation of population‐based hospitalization incidence rates. Countries generally agreed to collect the most basic data (tier 1) required to estimate proportions of hospitalization associated with RSV in the second phase of the surveillance. Countries with additional resources and capacities may opt to use more sophisticated approaches for estimation of RSV disease burden (Table 2) including the simple rapid assessment approach (method 5) to estimate the number of RSV‐related respiratory admissions. Regression modelling methods (method 1) will be possible once an adequate time series of these data has been collected, which will allow cross‐verification of the rapid assessment method. In addition, some study sites may be able to provide independent burden estimates at a later stage using one or more of the other methods (methods 2‐4) outlined in this paper, which will allow further cross‐validation.

**Table 1:** Tiered options to estimate RSV disease burden in the second phase of the WHO RSV surveillance

Tier	Burden estimate	Data source/variables required (cumulative by month)	Adjustment/correction factor	Preconditions to avoid bias	Caveats/limitations
Tier 0	None	Log of RSV positive Log of patients tested	None	None	‐ Can use ex‐SARI or SARI ‐ Weekly aggregation ‐ All‐year‐round surveillance
Tier 1.1	Proportion of respiratory or pneumonia hospital admissions due to RSV *(specify age‐band (0‐1y, 0‐2y)*	Log of RSV positive Log of patients tested Log of patients screened (for enrolment) Log of admissions by respiratory or pneumonia diagnosis (for non‐enrolment)	‐ Adjust for non‐enrolment ‐ Adjust for weekends or days of non‐enrolment	‐ Systematic sampling of enrolment days in week ‐ Systematic sampling of patients	‐ Adjustment factor for non‐enrolment estimated during season may overestimate burden during off‐season period ‐ Assumes that % positivity of RSV to be the same in those enrolled and those not enrolled ‐ Rrelationship between no. of extended‐SARI cases and no. of resp. or pneumonia cases may vary by season ‐ Burden estimate biased if sampling strategy is non‐random
Tier 1.2	Proportion of all‐cause hospital admissions due to RSV *(specify age‐band (0‐1y, 0‐2y)*	Log of RSV positive Log of patients tested Log of patients screened (for enrolment) Log of admissions (all‐cause) diagnosis (for non‐enrolment)	‐ Adjust for non‐enrolment ‐ Adjust for weekends or days of non‐enrolment	‐ Systematic sampling of weekdays ‐ Systematic sampling of patients	‐ Adjustment factor for non‐enrolment estimated during season may overestimate burden during off‐season period ‐ Assumes that % positivity of RSV to be the same in those enrolled and those not enrolled ‐ Burden estimate biased if sampling strategy is non‐random
Tier 2.1	RSV hospitalization rate per 100,000 pop. *(specify age‐band (0‐1y, 0‐2y)*	Log of RSV positive Log of patients tested Log of patients screened (for enrolment) Log of admissions by resp. or all‐cause diagnosis (for non‐enrolment) Catchment pop. Log of patients with resp. or all‐cause illness from catchment pop. that are admitted in non‐sentinel hospitals	‐ Adjust for non‐enrolment ‐ Adjust for weekends or days of non‐enrolment ‐ Adjust for patients with resp. illness from catchment pop. that seek care from other hospitals (Healthcare Utilization Survey data)	‐ Systematic sampling of weekdays ‐ Systematic sampling of patients ‐ Secondary‐level hospital with defined catchment pop. ‐ Healthcare admissions survey or healthcare utilization survey	‐ Based on WHO influenza disease burden estimation method ‐ Adjustment factor derived during season may overestimate burden during off‐season period ‐ Burden estimate biased if sampling strategy is non‐random ‐ HUS/HAS data required
Tier 2.2	Proportion of all‐cause ICU admissions due to RSV *(specify age‐band (0‐1y, 0‐2y)*	Log of ICU patients that are RSV positive Log of ICU patients tested Log of ICU patients screened (for enrolment) Log of ICU patients for all‐cause diagnosis (for non‐enrolment)	‐ Adjust for non‐enrolment ‐ Adjust for weekends or days of non‐enrolment	‐ Systematic sampling of weekdays ‐ Systematic sampling of patients	‐ Adjustment factor for non‐enrolment estimated during season may overestimate burden during off‐season period ‐ Assumes that % positivity of RSV to be the same in those tested and those not tested ‐ Assumes no significant bias in selection of patients for testing ‐ Burden estimate biased if sampling strategy is non‐random
Tier 2.3	Case fatality ratio *(specify age‐band (0‐1y, 0‐2y)*	Log of RSV positive Log of patients tested Log of patients screened (for enrolment) Log of patients by resp. or all‐cause diagnosis (for non‐enrolment) Log of RSV deaths	‐ Adjust for non‐enrolment ‐ Adjust for weekends or days of non‐enrolment	‐ Systematic sampling of weekdays ‐ Systematic sampling of patients	‐ Adjustment factor derived during season may overestimate burden during off‐season period ‐ Assumes that % positivity of RSV to be the same in those tested and those not tested ‐ Assumes no significant bias in selection of patients for testing ‐ Burden estimate biased if sampling strategy is non‐random ‐ Need to follow up RSV‐positive cases till discharge to determine death
Tier 2.4	Proportion of respiratory or pneumonia or all‐cause hospital deaths due to RSV *(specify age‐band (0‐1y, 0‐2y)*	Log of RSV positive Log of patients tested Log of patients screened (for enrolment) Log of patients by resp. or pneumonia or all‐cause diagnosis (for non‐enrolment) Log of RSV hospital deaths Log of resp. or pneumonia or all‐cause hospital deaths	‐ Adjust for non‐enrolment ‐ Adjust for weekends or days of non‐enrolment	‐ Systematic sampling of weekdays ‐ Systematic sampling of patients	‐ Adjustment factor derived during season may overestimate burden during off‐season period ‐ Assumes that % positivity of RSV to be the same in those tested and those not tested ‐ Relationship between no. of ex‐SARI cases and no. of resp. or pneumonia cases may vary by season ‐ Burden estimate biased if sampling strategy is non‐random ‐ Need to follow up RSV‐positive cases till discharge to determine death
Tier 3.1	National estimate of RSV hospitalization rate per 100,000 pop. *(specify age‐band (0‐1y, 0‐2y)*	Log of RSV positive Log of patients tested Log of patients screened (for enrolment) Log of admissions by diagnosis (for non‐enrolment) Census data: Mid‐year pop. by specified age bands, by administrative division serving hospital Adjusted for pop. increase Adjusted for the years of surveillance DHS data: To adjust admin division estimates to pop. estimates Pneumonia or influenza rates or prevalence of risk factors (HIV, malnutrition, crowding, prematurity etc) by region HUS data (if available): To adjust for non‐medically attended resp. illness	‐ Adjust for non‐enrolment ‐ Adjust for weekends or days of non‐enrolment ‐ Adjust for referrals from outside catchment population ‐ Adjust for non‐medically attended illness (optional)	‐ Systematic sampling of weekdays ‐ Systematic sampling of patients ‐ Secondary‐level hospital with defined catchment pop.	‐ Based on method described by Murray 2015, Theo 2017 ‐ Census data required ‐ DHS data on prevalence of certain morbidities required ‐ HUS data optionally required if adjustment for non‐medically attended illness ‐ Adjustment factor derived during season may overestimate burden during off‐season period

**Table 2:** Data points that will be needed to achieve a rapid assessment national burden estimate

Population data in age groups	Adjustment factors for population increase	Enrolled cases by case definitions	Screened to enrolled by case definition	Adjustment for non‐enrolment (weekends etc)	Adjustment for case referred into hospital	HUS* adjustment (if available)	Detection rate of RSV	DHS for provincial national estimates
Population of catchment area of hospital	Usually available from census, then able to adjust population for growth, may differ by hospital	Enrolled into surveillance, by each of the case definitions	#cases enrolled fitting case definition/#screened fitting case definitions	Adjustment factor for cases missed on days not enrolling like weekends. Logs of total admission during this time will need to be kept	Adjustment for cases referred in from outside catchment	Will account for cases who seek care at other hospitals	#RSV positive/total SARI (other case definitions)	Risk factors to compare hospital population to national population
Absolute number	Absolute number	Absolute number as per enrolment	#case/#screened = proportion				#RSV+/total SARI enrolled = proportion	Compare proportions of identified risk factors for pneumonia, apply adjustment of site is different from provincial or national

* Health utilization survey

The next phase of the WHO Global RSV surveillance programme will assist countries to better describe RSV health burden by collecting the necessary data needed. The critical data still to be collected in phase 2 of the WHO RSV surveillance are as follows: (a) documentation of total hospital admissions, respiratory admissions and patients screened but not enrolled into the surveillance programme and (b) the sampling fractions for cases in different time periods during the year. If countries opt for more sophisticated estimates of RSV disease burden, information will be additionally required on (a) what the catchment population is from which cases are drawn at each site (ie how many people would go to the surveillance facility if they developed symptoms of an RSV‐like illness), (b) the number of in‐referrals from outside the catchment area, (c) catchment population denominators and (d) Healthcare Utilization Surveys (to estimate the proportion of catchment population who would present at the sentinel facility for an acute respiratory illness).

The WHO RSV surveillance is making important progress to estimate RSV‐associated hospital‐related burden in a range of settings through the inclusion of additional data required to refine these estimates. The WHO candidate case definitions for severe and very severe RSV‐associated lower respiratory tract infections (LRTIs) require additional information on spO2, pulse oximetry and IMCI danger signs to be collected, as proxy indicators for disease severity.^17^ These disease severity indicators would allow trends in severe RSV disease burden to be monitored following the introduction of vaccines. Important steps remain to obtain other health‐related data to enable estimates of RSV‐related burden in primary care and for mortality. There remains much to be learnt from the experiences of measuring influenza burden, which could potentially be adapted for RSV.

## 7 CONCLUSIONS

Accurate in‐country disease burden estimates are important for local policy‐makers to make decisions on the introduction of prevention strategies and to employ cost‐effectiveness models when new RSV vaccines or monoclonal antibodies become available. Even simple estimates of proportion of hospitalizations associated with RSV are important to raise awareness of RSV disease burden among policy‐makers and providers. The WHO Global RSV surveillance programme aims to provide countries with a platform based on established approaches to collect these data. The next phase of the programme will assist countries with tools for collection of burden data, closely monitor sampling strategies for patient selection and testing, and strengthen the surveillance to provide simple, robust burden estimates for informed immunization policy decisions.
